# Effect of collimator and couch angle change on breast IMRT dose distributions

**DOI:** 10.1120/jacmp.v10i4.3058

**Published:** 2009-09-30

**Authors:** Jie Yang, Charlie Ma, Lu Wang, Lili Chen, Jinsheng Li

**Affiliations:** ^1^ Department of Radiation Oncology Penn State Cancer Institute Hershey PA 17033 USA; ^2^ Department of Radiation Oncology Fox Chase Cancer Center Philadelphia PA 19111 USA

**Keywords:** breast IMRT, dose distribution, Monte Carlo simulation

## Abstract

Intensity‐modulated tangential photon beams for breast cancer treatment can improve the dose uniformity significantly throughout the whole breast and reduce the dose to the lung and the heart when compared to the conventional technique. Before the first treatment, patient setup may require a change on the collimator angle and/or the couch angle based on the chest wall coverage according to the port films. The objective of this work is to investigate the effects of the collimator and the couch angle change on the dose distribution for breast cancer treatment using intensity‐modulated tangential photon beams, and to determine the clinical acceptable range of the angle change for routine treatment. Ten breast cases treated with intensity‐modulated tangential photon beams were analyzed in this study. Patient‐specific CT data and the radiation therapy planning (RTP) files obtained from our in‐house Monte Carlo based breast IMRT treatment planning system were used for IMRT dose recalculation with collimator or couch angle changes. The isodose distributions and DVHs were compared with the original plans, and the effects of the collimator and couch angle change to breast IMRT dose distributions were evaluated. Our results show that a 4° change in the collimator angle or the couch angle did not affect the dose distribution significantly and it is acceptable in the clinic for patient treatment.

PACS number: 87.10.Rt

## I. INTRODUCTION

Radiation therapy is demonstrated as one of the most effective modalities for breast cancer treatment, especially for early stage (T1‐T2) patients and breast conservation with lumpectomy. In radiotherapy, the conventional breast cancer management is to treat the whole breast using two wedged tangential photon beams with 45Gy to 50Gy, then the tumor bed is treated with an additional electron beam of 10Gy to 15Gy. A 90% to 95% local control rate and a 3%–5% or less complication rate has been achieved from the conventional technique.^(1)^ Some studies showed that intensity‐modulated radiation therapy (IMRT) using two opposed tangential photon beams for breast cancer can improve the dose uniformity throughout the breast and reduce the dose to the lung and the heart as compared with conventional techniques.^(^ ^2^ ^,^ ^3^ ^)^ Recently, a Monte Carlo‐based IMRT treatment planning system has been developed and used for breast treatment.^(4)^ In this planning system, an iterative method is used for optimization to generate IMRT plans and a step‐and‐shoot technique is used for beam delivery. The patient setup and incident beam directions are the same as those for conventional tangential photon treatment. The weights for the opposed beamlets in the two tangential beams are determined first by the doses at the depth‐of‐dose maximum at each side to minimize the hot spots. The intensity of an individual beamlet pair is then optimized based on the dose at the midplane. Over 200 breast patients have been treated with this planning system. The results show that the IMRT plan has similar dose coverage to the CTV as the conventional plan. The dose uniformity was improved significantly throughout the whole breast, especially at the inferior region. The doses to the lung and the heart were reduced and thus a reduction in the late effect was expected.

After an IMRT plan is generated, some port images are taken during the patient setup. A change to the collimator angle and/or couch angle may be required based on the chest wall coverage and edge matching to the supraclavicular field according to the port films. About 10% of breast patients required a less than 5° collimator or couch angle change in our clinic. The dosimetric effects and the clinically acceptable range of the angle change for routine breast treatment is unknown. The objective of this study is to investigate the effects of the collimator and the couch angle change on dose distribution, and to determine the clinically acceptable range of the angle changes for routine breast treatment.

## II. MATERIALS AND METHODS

The breast IMRT plans were generated using two opposite tangential fields. The fields were defined during the simulation such that a) the deep edges of the beams were coplanar (unopposed tangents); b) the beams covered the whole breast defined using the wires and markers; c) the beams were aligned such that the deep edges passed through the medial and lateral markers; d) the superficial edges of the beams allowed at least 2 cm of flash beyond the breast; e) the collimator was angled to accommodate the slope of the chest wall; f) cerrobend blocks were used and the couch was angled when it was needed to match the edge of a supraclavicular field which used an independent jaw setting; and g) symmetric jaw settings were used, so that the isocenter was placed in the middle of the fields. Once the treatment plan was approved by the physician, the patient's CT scan, treatment isocenter, leaf‐sequence files, beam parameters (gantry, table and collimator angle), and beam energy were retained for dose recalculation. Ten typical breast IMRT plans were chosen and analyzed in this study. 3D dose distribution in each patient was recalculated after the collimator angle or the couch angle was changed using the same dose calculation algorithm as that of the original plan. The collimator angles for both fields were changed accordingly, based on the tangential beam setting. The couch angle for one field was changed, while that of another one remained the same. The collimator angle and the couch angle were changed by 2°, respectively, for each calculation. The maximum collimator angle change and couch angle change were $\pm 8^{\circ }$, based on our routine clinical experience.

The dose calculation algorithm in the breast IMRT treatment planning system is the Monte Carlo method, which is a well‐known method for patient dose calculation. It can account for the effect of inhomogeneous anatomy and the beam delivery system more accurately than conventional methods. The Monte Carlo code used for breast IMRT dose calculation in this work was MCSIM,^(5)^ which is an EGS4/PRESTA user code with several variance reduction techniques activated.^(6)^ For all the simulations in this work, the electron and photon energy cutoffs for particle transport were set as ECUT=700keV ECUT=10keV, respectively. The energy thresholds for δ ray production (AE) and bremsstrahlung production (AP) were set as 700 keV and 10 keV, respectively. The maximum fractional energy loss per electron step (ESTEPE) was set to 0.04 and the default parameters were used for the PRESTA algorithm. The statistical uncertainty of Monte Carlo simulations was less than 1% (1 standard deviation) for all the calculations.

The differences in isodose distributions and dose‐volume histograms (DVHs) were compared between the plans with angle changes and the original breast IMRT plans. The clinical target volume (CTV) was defined to be the entire breast tissue as delineated on the CT data set. Superficially, the CTV was extended to within 5 mm of the skin surface. The treatment fields were extended 2 cm beyond the CTV in the superior and inferior direction. A flash was included in the treatment fields. The percentage volumes of CTV receiving 95% (V95), 105% (V105) of the prescription dose and the dose received by 95% volume of CTV (D95) were compared with those of the original plan. Some clinical criteria for critical structures, such as the percentage volume of the ipsilateral lung receiving 20Gy (V20) and the percentage volume of heart receiving 30Gy (V30), were also compared with those of the original plan after the collimator angle or couch angle was changed. Based on the preliminary results, we have established clinical criteria to accept an IMRT plan for breast treatment in which: (1) V95 for the CTV must be higher than 95%; (2) V100 for the CTV must be higher than 85%; (3) V105 for the CTV must be less than 10%; (4) maximum dose must be less than 110% of the prescription dose; (5) V20 for the ipsilateral lung must be less than 15%; and (6) V30 for the heart must be less than 1%. If the recalculated results with angle change meet the clinical criteria and the differences between the recalculated dose distribution and the original plan are not significant, the angle change is considered as clinically acceptable. Based on the evaluation results, the clinically acceptable range of collimator angle and couch angle change can then be determined. The range is the maximum allowed angle adjustment during patient setup.

## III. RESULTS

**Figure 1:**
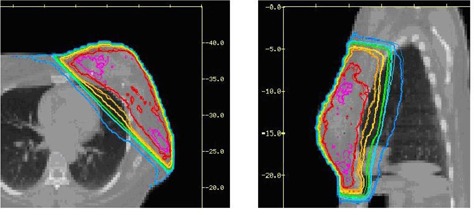
Isodose distribution comparisons in the axial and sagittal view. The thick line is for the original plan and the thin line is for the plan with a couch angle change.

**Figure 2:**
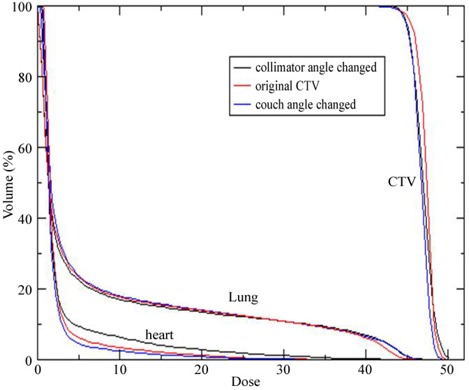
DVH comparison between the original plan and the plan with collimator or couch angle changes.

**Figure 3:**
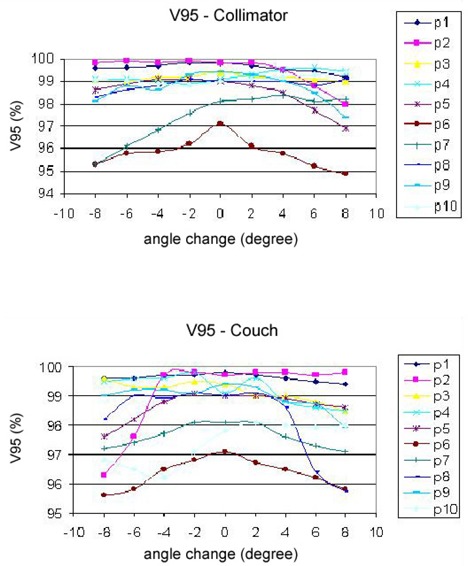
V95 comparisons for the 10 breast IMRT plans between the original plan and the plan with angles changed.

**Figure 4:**
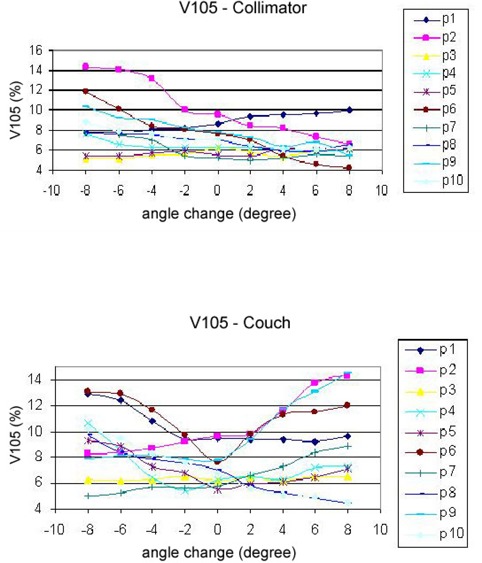
The effect of couch or collimator angle changes on V105 of CTV for 10 plans.

**Figure 5:**
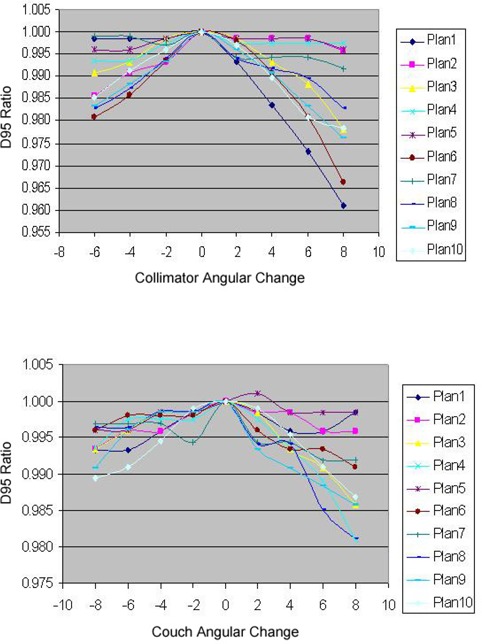
D95 ratio as a function of collimator or couch angle change for the 10 breast IMRT plans.

**Figure 6:**
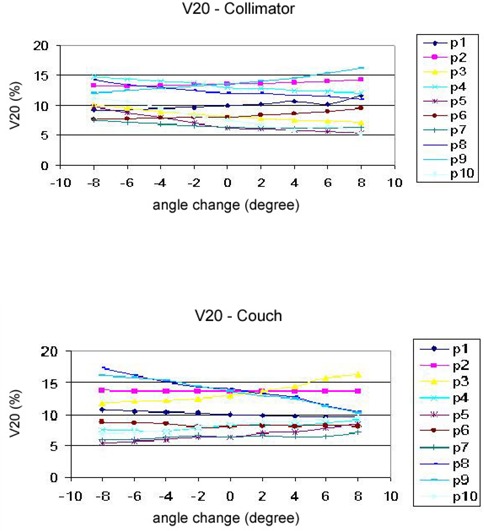
Comparison of V20 for the lung when collimator or couch angles were changed.

**Figure 7:**
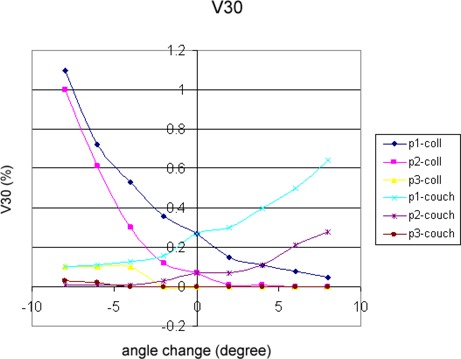
V30 comparison for the heart between the original plan and the plan with collimator or couch angle changes.

3D dose distributions of 10 breast IMRT plans have been evaluated with collimator angle or couch angle change. The DVHs and isodose distributions were compared between the recalculated results with angle change and their respective original plans. Figure 1 shows the comparison of isodose distributions after a 6° couch angle change in the axial and sagittal view, and no significant difference was observed except for those in the low dose region. Figure 2 shows DVH parameters for the original plan, plan with 6° collimator angle change and plan with 6° couch angle change. Differences on CTV dose and critical structure dose can be observed. In order to show the effects of angle change quantitatively, V95, V105, and D95 of CTV for the 10 breast IMRT plans were compared with different collimator or couch angle change. Figure 3 shows the comparison of V95 for the 10 breast IMRT plans. V95 of CTV for all the plans were still in the acceptable range (> 95%) based on our clinical criteria when collimator angle or couch angle changed from −8° to 8°. The effect of angle change on the V105 of CTV is shown in Fig. 4. Bigger hot spots can be observed for some patients when the collimator or couch angle change is more than 4°. However V105 is still less than 10% for most of the cases. Figure 5 shows the ratio of D95 relative to the original plan after the collimator or couch angle was changed. The effects on D95 are less than 2% for most of the cases, especially when the angle change is less than 4°. Different effects caused by the angle changes can be observed for different patients because of the different breast shape. Collimator angle or couch angle change also has some effects on the dose of critical structures, such as the lung and the heart. Figure 6 is the comparison of V20 for the lung when collimator or couch angle changed. The effect on V20 of the lung was less than 4% if the angle change is less than 4°. In only one case was the difference on V20 larger than 10% when couch angle changed 8°, but differences on V20 for almost all the cases were larger than 10% when collimator change was 8°. In our study, there were three left breast cases in which the heart was considered as the critical structure because of its anatomical presence. V30s of the heart for all three plans were compared and the results are showed in Fig. 7. All the new plans for the three patients are still acceptable based on our criteria when the angle changes were less than 6°, and the V30 was more sensitive to the collimator angle change than to the couch angle change. We found that both CTV and critical structures were more sensitive to the collimator angle change than to the couch angle change.

## IV. CONCLUSIONS

Effects of collimator or couch angle change on the dose distribution have been evaluated for 10 breast IMRT plans. Our results show that the effects on isodose distribution were not significant when collimator or couch angle changes were less than 6°. There was no significant difference on DVH of the CTV if the collimator angle or the couch angle change was less than 4°. Based on the results for critical structures, up to 4° on the angle change could be allowed for the lung, and up to 6° on the angle change could be allowed for the heart. Overall, up to 4° change on the collimator or couch angle from the initial simulation is permissible for breast patient treatment using tangential IMRT.
